# Clinical improvement of sepsis by extracorporeal centrifugal leukocyte apheresis in a porcine model

**DOI:** 10.1186/s12967-022-03752-6

**Published:** 2022-11-22

**Authors:** Lei Zhou, Dong Zhang, Ling Kong, Xiaodong Xu, Dehua Gong

**Affiliations:** National Clinical Research Center of Kidney Diseases, Jinling Hospital, Nanjing University School of Medicine, 305 Zhongshan East Road, Xuanwu District, Nanjing, 210016 China

**Keywords:** Sepsis, Extracorporeal centrifugal leukocytapheresis, Inflammation, Immune modulation

## Abstract

**Background:**

Extracorporeal blood purification therapies targeting removal of the downstream products of the inflammatory cascade in sepsis have failed to improve mortality. As an upstream process of the inflammatory cascade, activated white blood cells should be a potential therapeutic target for sepsis, and the effect of removing such cells by extracorporeal centrifugal leukocytapheresis (LCAP) is worth considering.

**Methods:**

Fourteen peritonitis-induced septic pigs were randomly assigned to receive a sham operation (control group, n = 7) or one session of LCAP at 12 h after sepsis induction (treatment group, n = 7). Samples from peripheral blood at various time-points and from LCAP collection were tested. All pigs were euthanized at 48 h, and lung, kidney, liver and spleen tissues were obtained for histopathological examination.

**Results:**

Two pigs died in accidents before the induction of sepsis, and 12 pigs were finally included for the statistical analysis. A significant clinical improvement was present in the treatment group relative to the control group in terms of the mean arterial blood pressure (MAP), oxygen tension (PaO_2_), lactic acid level, oxygenation index (PaO_2_/FiO_2_), and carbon dioxide tension (PaCO_2_, P < 0.05). Flow cytometry tests showed that a mixture of B cells, dendritic cells, T helper cells, cytotoxic T cells, monocytes and neutrophils were removed from the circulation by LCAP, resulting in sepsis-induced change trends in the control cells; these change trends were all flattened in the treatment group, although nonsignificantly.

**Conclusions:**

LCAP may exert a wide-spectrum and bidirectional immunomodulatory effect on sepsis, accompanied by improvements in hemodynamics and oxygenation status.

## Introduction

Although there has been substantial progress in the management of sepsis recently, it is still a great challenge for the health care system in high-income countries, with a reported annual incidence of 31.5 million affected cases and 5.3 million deaths, which is a much higher mortality rate than that of other diseases [1, 2]. This fact facilitates perpetual efforts in exploring novel methods for the treatment of sepsis. According to the current knowledge, a dysregulated host immune response to infection is considered the key underlying pathophysiological cause for life-threatening organ dysfunction presented in sepsis. [3, 4] Therefore, methods aimed at modulating immune function have become a research approach to seeking novel treatment options for sepsis. There have been numerous investigations of the role of extracorporeal blood purification therapies in the treatment of sepsis through the removal of proinflammatory substances such as cytokines and endotoxins from blood (reviewed in [5]). Such therapies may be proposed as adjuncts to standard treatments (such as antibiotics, management of organ dysfunction, and surgical treatment as required). As reported, these methods include high-volume hemofiltration (HVHF) [6], cascade hemofiltration [7], hemoperfusion or plasma perfusion using endotoxins/cytokine adsorption devices such as Toraymyxin^®^ (polymyxin B-immobilized fiber column), [8] CytoSorb^®^^[9]^, the AN69ST membrane [10], and oXiris^®^^[11]^. However, the effectiveness of these methods remains controversial, with most trials failing to prove their superiority over standard treatments [5]. The failure of methods targeting the removal of plasma toxic solutes should turn research interests to targeted intervention of immune cells, which are the major sources of cytokine production. Sepsis induces immune alterations in innate and adaptive cells [3], and marked elevation of white blood cells (WBCs) in circulation is a physical response to infection in the early stage of sepsis. Based on these considerations, it is worth investigating the effect of eliminating dysfunctional WBCs from the circulation during the early stage of sepsis using centrifugal cell separation techniques such as leukocytapheresis (LCAP). To address this issue, we performed a randomized controlled experimental study to explore the effect of LCAP on immune modulation and improvement of clinical conditions in a peritonitis-induced septic porcine model.

## Methods

All animal experiments were performed in the Department of Comparative Medicine, Jinling Hospital, with a protocol previously approved by the Institutional Animal Care and Use Committee of Jinling Hospital, Nanjing University (grant number 2017NZGKJ-019). The treatments of animals were fully in compliance with the National Research Council’s 1996 Guide for the Care and Use of Laboratory Animals [12].

### Animal preparation

Fourteen domestic healthy sex-unspecified pigs with weights of 50–60 kg were fed in the Department of Comparative Medicine 1 week prior to the study and were fasted except for free access to water for 8 h just before the study. At the start of the study, the pigs were anesthetized by intramuscular injection with a mixture of midazolam (2 mg/kg), atropine (1.0 mg) and diazepam (20 mg), followed by a continuous infusion of 2% propofol (4 mg/kg/h) through the ear vein as maintenance anesthesia. Thereafter, the pigs received an intraperitoneal injection of a suspended solution obtained by incubation of previously collected autologous feces (1.0 g/kg) with 200 ml sterile saline at room temperature (25–30 °C) for 8 h. In the following 12 h, the pigs were transferred to the animal intensive care unit without constraints, and those with an increase of  ≥ 2 sequential organ failure assessment (SOFA) points, which included assessment of respiratory, hematological, circulatory, nervous, digestive and urinary systems [4, 13], were considered successful in the induction of sepsis. Then, the pigs were anesthetized again. Tracheal intubation with no mechanical ventilation was performed to keep the airways open. Basal fluid support (7 ml/kg/h) was given using the hospital-made balanced solution (Na^+^ 142.5 mmol/L, K^+^ 3.72 mmol/L, Ca^2+^ 1.5 mmol/L, Mg^2+^ 1.56 mmol/L and glucose 11.0 mmol/L, HCO3^−^34 mmol/L) (Patent Number: CN1338316A) [14–16]. Vasopressors (such as norepinephrine and dopamine) and fluid resuscitation were used to stabilize the arterial pressure if necessary. A dual-lumen catheter (12F 12.5 cm, BARD, U.S. A) was inserted into the right jugular vein for vascular access.

After the induction of sepsis, all the pigs were randomly assigned to the control group and the treatment group at a ratio of 1:1. The control group received only fluid infusion and vasopressors if necessary, while the treatment group received one session of extracorporeal centrifugal LCAP in addition to the therapies given in the control group.

### Extracorporeal centrifugal leukocytapheresis therapy

**Fig. 1:**
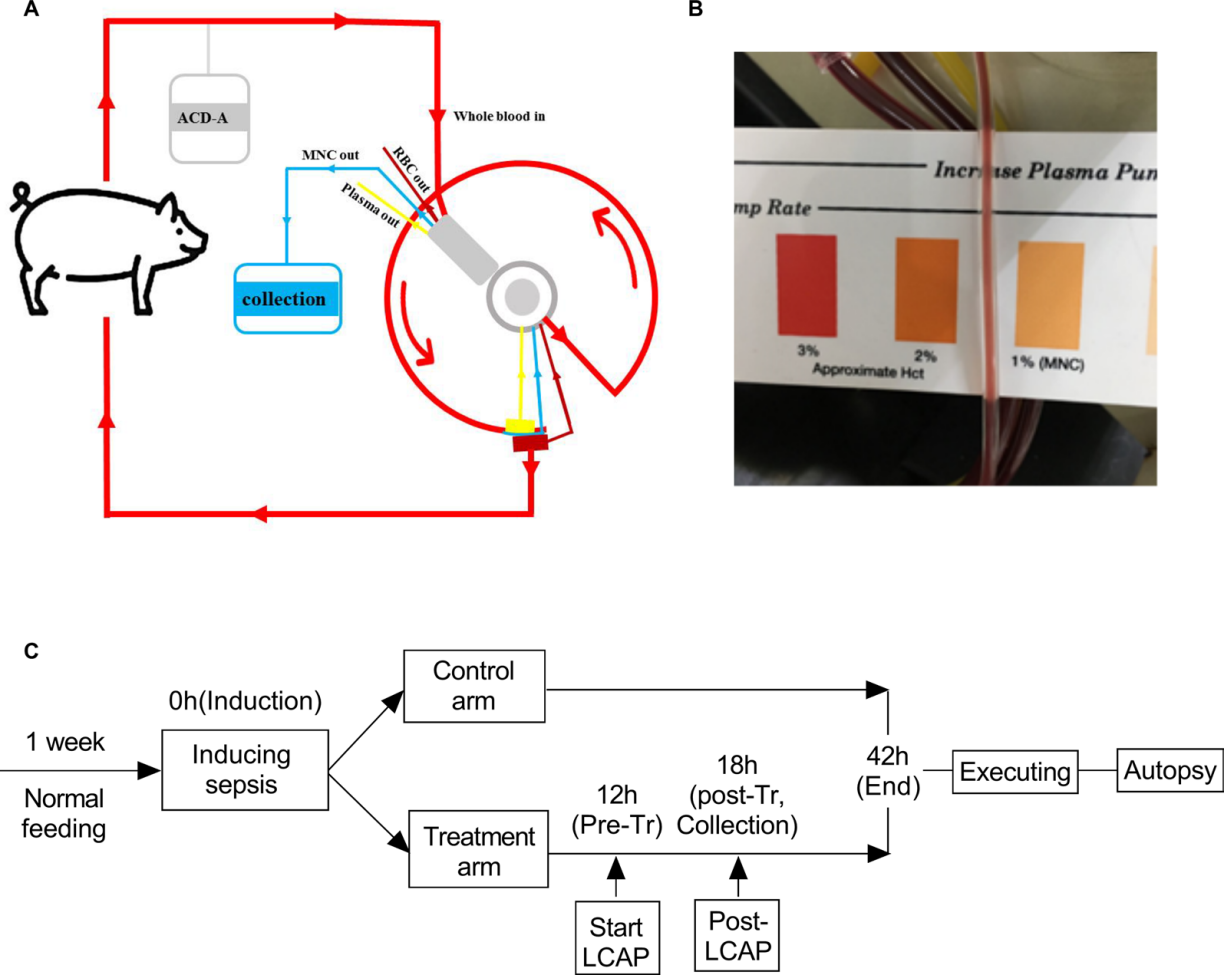
The schema and flow diagram of extracorporeal centrifugal leukocyte apheresis therapy. **A** The pig was connected to the centrifugal system and received cellular apheresis therapy. **B** The collection was compared to the colorimetric card to adjust the collect/replace pump flow. **C** After induction of sepsis, the control arm received only fluid therapy and inotropic drugs if necessary, while the treatment arm received one session of LCAP in addition to the control arm treatments. Induction, Pre-Tr, Post-Tr, and End represent blood sampling at this timepoint; Collection represents sampling from the harvesting products after LCAP in the treatment group. LCAP, leukocytapheresis

In the treatment group, LCAP started 12 h after intraperitoneal injection of the feces-suspended solution. The LCAP procedures followed the instructions of the “MNC” program provided by the manufacturer of the Cobe Spectra Apheresis System (CaridianBCT, Collins Avenue, Lakewood, Colorado 80215, U.S.A.). This program mainly harvests peripheral mononuclear cells from the circulation through a centrifugal cell separation technique. The blood flow rate was set at 40–60 ml/min, with anticoagulation by infusion of acid citrate dextrose solution A (ACD-A) at a blood flow rate ratio of 1:10–1:15 (Fig. 1A). The rate of the cell harvesting pump was set at 1 ml/min, with adjustment based on the color of the harvesting fluid according to a colorimetric card provided by the manufacturer (Fig. 1B). Each session lasted 1–1.5 h, with two times the total blood volume (TBV) processed.

### Monitoring and sampling

All pigs were euthanized by injection 42 h after induction of sepsis. Vital signs were monitored throughout the whole period of the experiment. Data and blood sampling were collected at baseline, 12 h after induction of sepsis, 42 h after induction of sepsis in all pigs, and after LCAP in the treatment group (Fig. 1C). Samples from the harvested product were also collected for further measurements. Blood cell analysis, indices of hepatic and renal function, arterial blood gas analysis and endotoxin levels were tested in the Clinical Laboratory of Jinling Hospital. Endotoxin levels were tested by a tachypleus amoebocyte lysate assay. C-creative protein (CRP), procalcitonin (PCT), and cytokines (IFN-γ, TNF-α, IL-6, IL-10) were tested by a ELISA kit (Elabscience Biotechnology Co., Wuhan, China) following the manufacturer’s instructions. Myeloperoxidase (MPO) activity was measured by the method described by S Fietz et al. [17].

Cell typing was performed via flow cytometry using various leukocyte surfaces, as follows: monocytes by CD14 (Abcam, ab186689)and CD163 (AbD Serotec, MCA2311F), B cells by CD21 (Abcam, ab65809), dendritic cells by CD3^−^CD172a^+^ (AbD Serotec, MCA1222A647 and MCA2312F), T helper (Th) cells by CD3^+^CD4^+^ (AbD Serotec, MCA1222A647 and MCA1749F), cytotoxic T (Tc) cells by CD3^+^CD8^+^ (AbD Serotec, MCA1222A647 and Abcam, ab25536), and activated neutrophils by CD113R (AbD Serotec, MCA2309F).

### Histopathology

At the end of the experiment, the pigs were euthanized through rapid intravenous injection of propofol until respiratory and cardiac arrest. Lung, kidney, liver and spleen specimens obtained after euthanasia were fixed with 10% formalin for histopathological examination. Hematoxylin–eosin staining was performed routinely, and immunohistochemistry was also performed using antibodies specific for anti-CD11b [EPR1344] (Abcam, ab133357) and SIRPA/CD172a (clone BL1H7) (LifeSpan, LS-C187488). Relative gray values of positive tissues were calculated. Two independent pathology professionals blindly evaluated and scored the severity of the lesions as follows: 0, normal; 0.5, slight or very small lesions; 1, mild or small lesions; 2, moderate or common lesions; 3, severe or diffusive lesions; and 4, extremely severe or massive lesions [18].

## Calculations

$$ {\text{Females}}:{\text{ TBV in ml }} = { 183 } + \, \left[ {{356} \times {\text{height}}^{{3}} \left( {\text{m}} \right)} \right] \, + \, \left[ {{33}.{1} \times {\text{weight }}\left( {{\text{kg}}} \right)} \right] $$

$$ {\text{Males}}:{\text{ TBV in ml }} =\, { 6}0{4 } + \, \left[ {{367} \times {\text{ height}}^{{3}} \left( {\text{m}} \right)} \right] \, + \, \left[ {{32}.{2} \times {\text{weight }}\left( {{\text{kg}}} \right)} \right] $$

$$ {\text{Total leukocyte count }} = {\text{ leukocyte concentration}}\left( { \times {1}0^{{9}} /{\text{L}}} \right) \, \times {\text{TBV }}\left( {{\text{mL}}} \right)/{1}000 $$

$$ {\text{Total collected leukocyte count }} = {\text{ collected leukocyte concentration }}\left( { \times {1}0^{{9}} /{\text{L}}} \right) \times {\text{ collectionvolume }}\left( {{\text{mL}}} \right)/{1}000 $$

### Statistical analysis

Means ± standard deviations are presented for normally distributed continuous variables, and medians (interquartile ranges) are presented for skewed continuous variables. The Shapiro‒Wilk test was used to assess the normality of the variables. The independent-samples T test was used to compare normally distributed parameters, and a nonparametric test was used to compare skewed parameters of the control group and treatment group. The differences in normally distributed parameters among time points (≥ 3 time points) in each group were compared by one-way ANOVA, and the least significant difference (LSD) test was used for the post hoc test. The differences in skewed parameters among time points (≥ 3 time points) in each group were compared by nonparametric tests and post hoc tests for multiple comparisons. Data analyses were performed by SPSS 21.0 software, and graphs were processed by GraphPad Prism 6 software. Statistical significance was set at P < 0.05.

## Results

Two pigs died before the induction of sepsis, one because of a catheterization-associated accident and one because of an intubation-associated accident. Finally, 12 pigs were randomized equally into the control and treatment groups. All of them reached the criteria of sepsis [2] 12 h after induction, with blood LPS positivity, and survived to the end of the study, except one pig in the treatment group, which experienced resistant hypotension during LCAP and died 8 h after the end of treatment, with data included in the final analysis.

There were no significant differences in any measured data between the control group and treatment group at baseline (Table 1).

**Table 1:** Baseline variables between control and treatment groups*

	Control group	Treatment group	P value
Weight (kg)	54.4 ± 8.7	54.3 ± 11.3	0.995
Body length (cm)	125.5 (124.0, 128.5)	125.0 (124.0, 132.3)	0.937
TBV (L)	2.8 ± 0.5	2.8 ± 0.5	1
MAP (mmHg)	125.6 ± 10.1	130.5 ± 13.5	0.932
HR (beats/minute)	103 ± 11.1	105 ± 8.6	0.438
Blood cells			
Leukocyte (*10^9/L)	15.6 ± 1.5	14.2 ± 3.1	0.394
Hemoglobin (g/L)	104.0 ± 13.2	99.7 ± 12.4	0.611
Hct (%)	30.8 ± 4.4	33.6 ± 3.9	0.327
Platelet (*10^9/L)	365.75 ± 107.0	367.5 ± 138.0	0.984
B Cell (%)	3.6 ± 1.1	3.5 ± 1.5	0.947
DC Cell (%)	4.2 ± 1.5	4.2 ± 1.5	0.997
Th cell (%)	4.0 ± 2.7	6.9 ± 4.2	0.271
Tc cell (%)	7.5 ± 1.5	13.1 ± 5.6	0.093
Th/Tc cells (%)	51.9 ± 25.5	56.9 ± 36.8	0.822
Monocyte (%)	1.9 (1.6, 2.4)	2.3 (1.8, 4.2)	0.310
Neutrophil (%)	48.6 ± 3.8	42.6 ± 8.1	0.210
Hepatic and renal function			
ALT (U/L)	82.0 ± 64.2	105.0 ± 78.4	0.640
AST (U/L)	33.5 (28.0, 64.5)	36.5 (31.8, 60.5)	0.485
TBIL (μmol/L)	1.9 ± 1.1	1.5 ± 1.0	0.531
DBIL (μmol/L)	0.4 ± 0.2	0.3 ± 0.3	0.629
IBIL (μmol/L)	1.5 ± 1.0	1. 2 ± 0.8	0.562
Alb (g/L)	23.3 ± 6.3	24.0 ± 2.7	0.807
Creatine (μmol/L)	94.5 ± 19.2	91.5 ± 20.9	0.825
Urea (mmol/L)	3.1 ± 1.0	2.6 ± 0.6	0.359
Blood gas assay			
PH	7.5 ± 0.04	7.5 ± 0.02	0.543
PaO_2_ (mmHg)	99.0 (94.8, 113.3)	96.0 (86.5, 101.5)	0.310
PaCO_2_ (mmHg)	47.0 ± 4.2	46.8 ± 3.8	0.949
PiO_2_/FiO_2_ (mmHg)	521.6 ± 83.3	447.6 ± 42.6	0.098
Lac (mmol/L)	1.6 ± 0.7	1.6 ± 0.2	0.868

^*^Both the control and treatment group were induced sepsis after baseline evaluation

*TBV* total blood volume, *MAP* mean arterial blood pressure, *HR* heart rate, *Hct* hematocrit, *DC cell* dendritic cell, *Th cell* T helper cell, *Tc cell* cytotoxic T cell, *ALT* alanine transaminase, *AST* aspartate aminotransferase, *TBIL* total bilirubin, *DBIL* direct bilirubin, *IBIL* indirect bilirubin, *Alb* albumin, *PH* pondus hyrogenil, *PaO*_*2*_ partial pressure of arterial oxygen, *PaCO*_*2*_ partial pressure of arterial carbon dioxide, *SaO*_*2*_ oxyhemoglobin saturation, *Lac* lactate

### Clinical and histology findings

The systemic hemodynamics and oxygenation status are summarized in Table 2. In contrast to the control group, improvements in clinical conditions were found in the intervention groups, manifesting as significantly a higher MAP, PaO_2_, and PaO_2_/FiO_2_ and a lower heart rate, lactic acid level and PCO2 (P < 0.05). These results indicate that LCAP therapy improved alveolar ventilation and maintained hemodynamic stability. The liver and kidney functions did not change significantly in either group (data not shown).

**Table 2:** Parameters of systemic hemodynamics and oxygenation state between control and treatment groups*

		Baseline	12 h	18 h	42 h	P’value
MAP (mmHg)	Control	125.6 ± 10.1^b^	110.3 ± 17.6	93.2 ± 11.3	85.3 ± 16.3	0.015
	Treatment	130.5 ± 13.5^a^	101.2 ± 13.7^c^	99.2 ± 20.5	110.3 ± 9.5	0.049
	P value	0.932	0.297	0.053	0.031	–
HR (beats/minute)	Control	103 ± 11.1^ab^	112.6 ± 14.5	109.7 ± 20.3	130.6 ± 15.6	0.001
	Treatment	105 ± 8.6^a^	120.1 ± 6.2^c^	123.5 ± 12.5	100.3 ± 23.7	0.049
	P value	0.438	0.683	0.044	0.015	–
PH	Control	7.5 ± 0.04	7.6 ± 0.04	–	7.5 ± 0.03	0.064
	Treatment	7.5 ± 0.02	7.5 ± 0.08	7.5 ± 0.03	7.5 ± 0.03	0.138
	P value	0.543	0.021	–	0.551	–
PaO2 (mmHg)	Control	99.0 (94.8, 113.3)^ab^	87.0 (77.5, 94.0)	–	74.0 (69.5, 78.3)	0.025
	Treatment	96.0 (86.5, 101.5)^a^	75.5 (64.8, 82.8)^c^	77.5 (72.3, 83.8)	95.5 (92.3, 96.3)	0.011
	P value	0.310	0.092	–	0.003	–
PaO_2_/FiO_2_	Control	521.4 ± 83.3^ab^	390.3 ± 38.7	–	352.3 ± 20.9	0.000
	Treatment	447.6 ± 42.4^a^	381.3 ± 50.7^c^	369.1 ± 39.6	450 ± 12.3	0.003
	P value	0.098	0.779	–	0.000	–
PaCO2 (mmHg)	Control	47.0 ± 4.2^a^	45.0 ± 1.4^c^		48.2 ± 1.7	0.049
	Treatment	46.8 ± 3.8^b^	46.3 ± 6.2	46.0 ± 2.4	41.5 ± 2.1	0.003
	P value	0.949	0.051	–	0.001	–
Lac (mmol/L)	Control	1.6 ± 0.7^ab^	2.9 ± 0.5	–	3.1 ± 1.3	0.034
	Treatment	1.6 ± 0.2^a^	3.3 ± 1.5^c^	1.4 ± 0.4	1.2 ± 0.3	0.003
	P value	0.868	0.112	–	0.008	–

^*^Both the control and treatment group were induced sepsis after baseline evaluation

*MAP* mean arterial blood pressure, *HR* heart rate, *PH* pondus hyrogenil, *PaO*_*2*_ partial pressure of arterial oxygen, *PaCO*_*2*_ partial pressure of arterial carbon dioxide, *Lac* lactate

P < 0.05 stands for statistical significance between control and treatment groups

P’ < 0.05 stands for statistical significance within every time points at each group

^a^stands for statistical significance between baseline and 12 h

^b^stands for statistical significance between baseline and 42 h

^c^statistical significance between 12 and 42 h through post hoc test

**Fig. 2:**
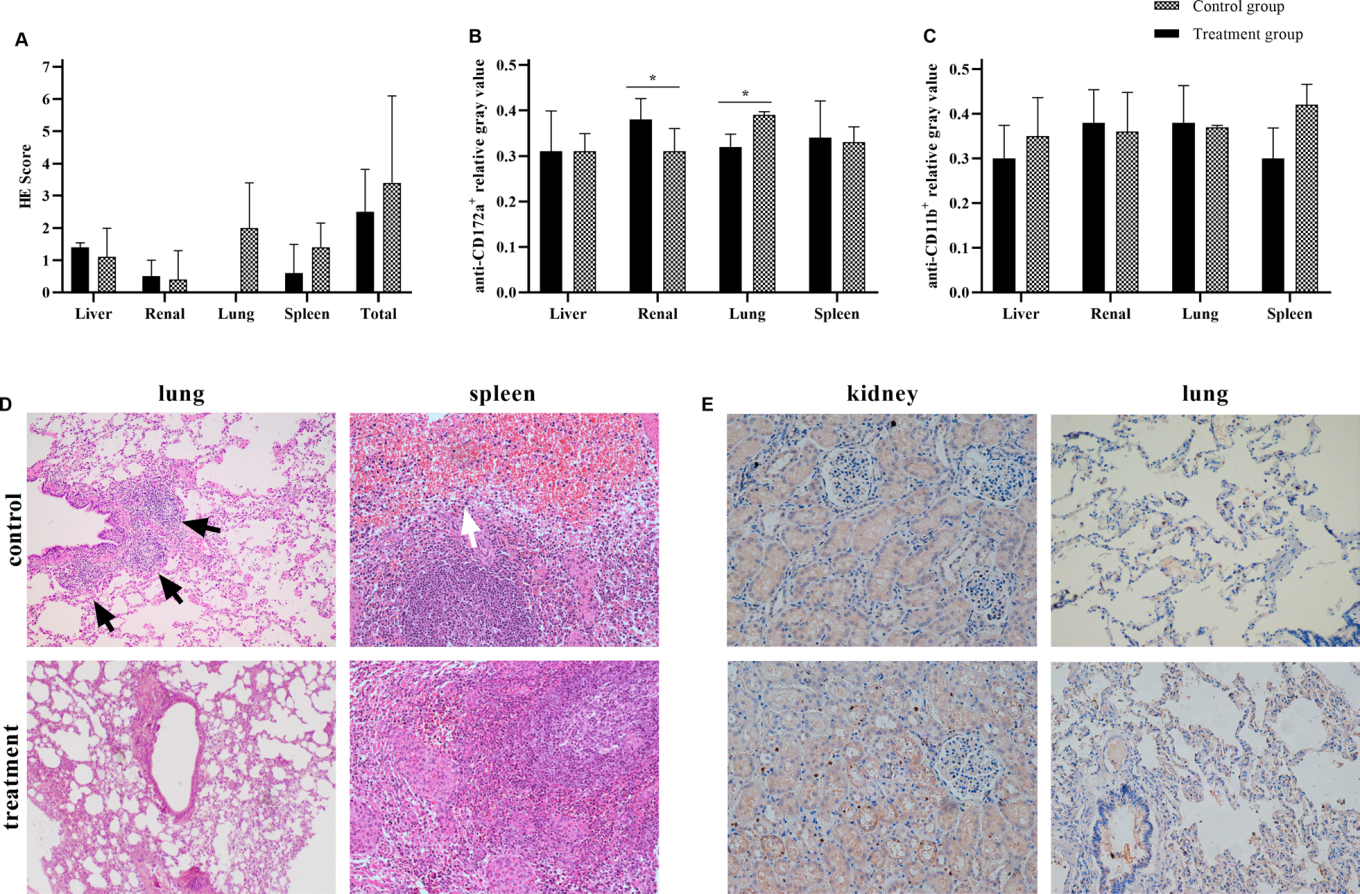
Organ injury and immune cell infiltration. **A** and **D** The degree of organ lesions; **B** and **E** CD172^+^ cell infiltration in organs; **C** CD11b^+^ cell infiltration in organs. The (*) stands for statistical significance between the two groups (P < 0.05). Black arrows, lymphoid follicles; white arrow, congested red pulp

Histological examination showed no significant difference in the degree of organ lesions between the two groups (2.5 ± 1.32 vs. 3.4 ± 2.7, P > 0.05) or the lesion scores of each organ, but more severe lesions of the lung (2.0 ± 1.4 vs. 0.0 ± 0.0) and spleen (0.6 ± 0.9 vs. 1.4 ± 0.8) were found in the control group compared with the intervention group (Fig. 2A). In the control group, there were more lymphoid follicles around the bronchus of the lung, and more congestion in the splenic red pulp was detected. (Fig. 2D). CD172^+^ cell infiltration was more common in the kidneys (0.38 ± 0.05 vs. 0.31 ± 0.05, P = 0.049) but less common in the lungs (0.32 ± 0.03 vs. 0.39 ± 0.01, P = 0.016) of the intervention group compared to the control group (Fig. 2B, E). CD11b^+^ cell infiltration was not significantly different in any tissue between the groups (Fig. 2C).

### Changes in inflammatory cytokines

**Fig. 3:**
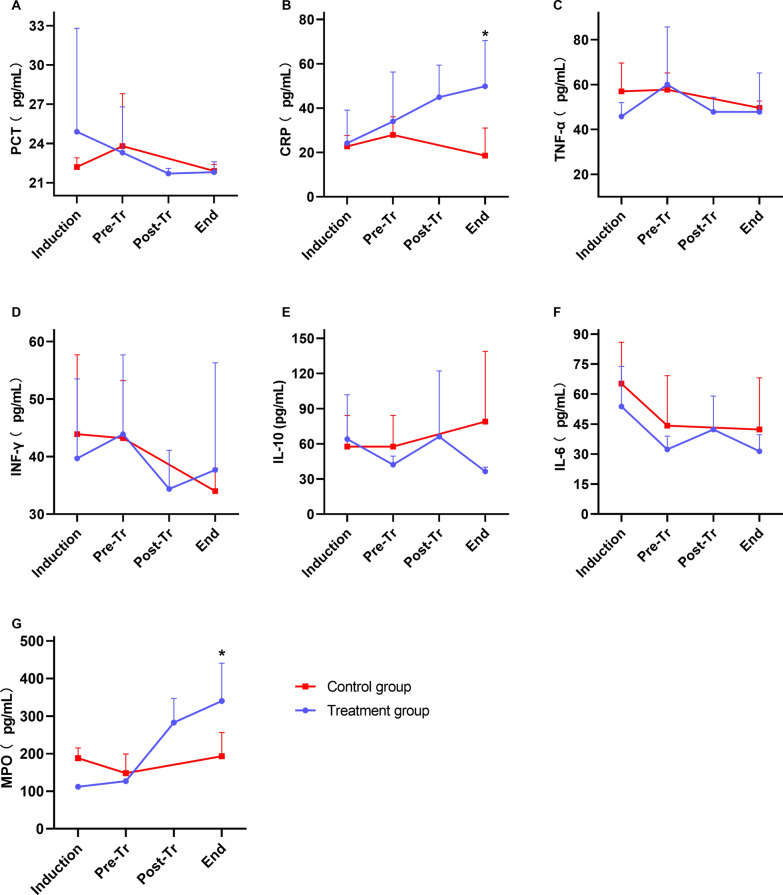
Changes in inflammatory cytokines. **A** PCT; **B** CRP; **C** TNF-α; **D** INF-γ; **E** IL-10; **F** IL-6; **G** MPO. The (*) stands for statistical significance between the two groups (P < 0.05). Abbreviations: PCT, procalcitonin; CRP, C reaction protein; MPO, myeloperoxidase. (Induction, baseline and sepsis induction; Pre-Tr, 12 h after sepsis induction and before treatment; Post-Tr, the end of treatment; End, 24 h after treatment and time before euthanasia)

The changes in plasma cytokine levels are shown in Fig. 3. After treatment, the levels of PCT, TNF-α, INF-γ and IL-6 were not different between the two groups; the CRP level and MPO activity of the treatment group were higher than those of the control group (P < 0.05), and IL-10 was lower in the treatment group, but the difference was not significant.

### Composition of the collections of LCAP and changes in peripheral blood cells

**Fig. 4:**
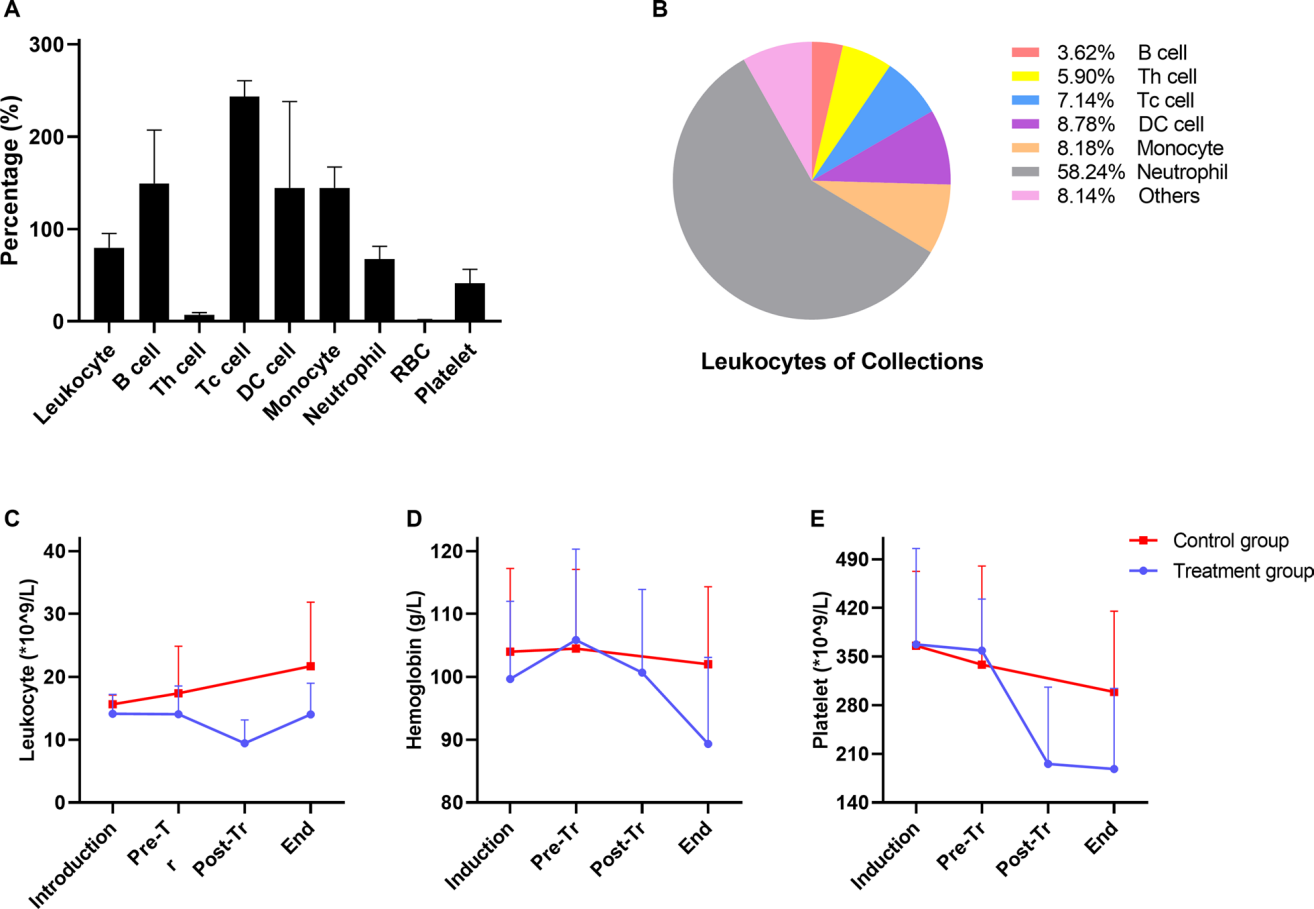
Composition of the collections and peripheral blood. **A** Cell counts in the collection are expressed as a percentage of pretreatment cell counts in the blood. **B** Constituent proportions in the collected leukocytes. **C**, **D** and **E** represent changes in leukocytes, hemoglobin and platelets in the peripheral blood. The (*) stands for statistical significance between the two groups (P < 0.05). (Induction, baseline and sepsis induction; Pre-Tr, 12 h after sepsis induction and before treatment; Post-Tr, the end of treatment; End, 24 h after treatment and time before euthanasia.)

The average volume of collections was 184 ± 120.7 ml. The leukocyte, RBC and platelet counts in the collection were 79.6%, 1.2%, and 41.1% of the pretreatment levels of blood, respectively (Fig. 4A). For leukocyte subgroups, the counts of Tc cells, B cells, DC cells and Th cells in the collection were 250%, 150%, 150%, and 6.9% of the pretreatment levels of blood, respectively. The constituent proportions in the collected leukocytes were as follows: B cells 3.62%, Th cells 5.90%, Tc cells 7.14%, DC cells 8.78%, monocytes 8.18%, and activated neutrophils 58.24% (Fig. 4B).

After the induction of sepsis, the pigs in the control group presented with a progressive increase in leukocyte count, a decrease in platelet count and a slight decrease in hemoglobin level, while in the intervention group, the leukocyte count declined post-LCAP and returned to the baseline level after 24 h, accompanied by a significant decrease in hemoglobin level and platelet count (Fig. 4C, Dand E). However, there was no statistically significant difference between the two groups.

### Changes in subgroups of leukocytes

**Fig. 5:**
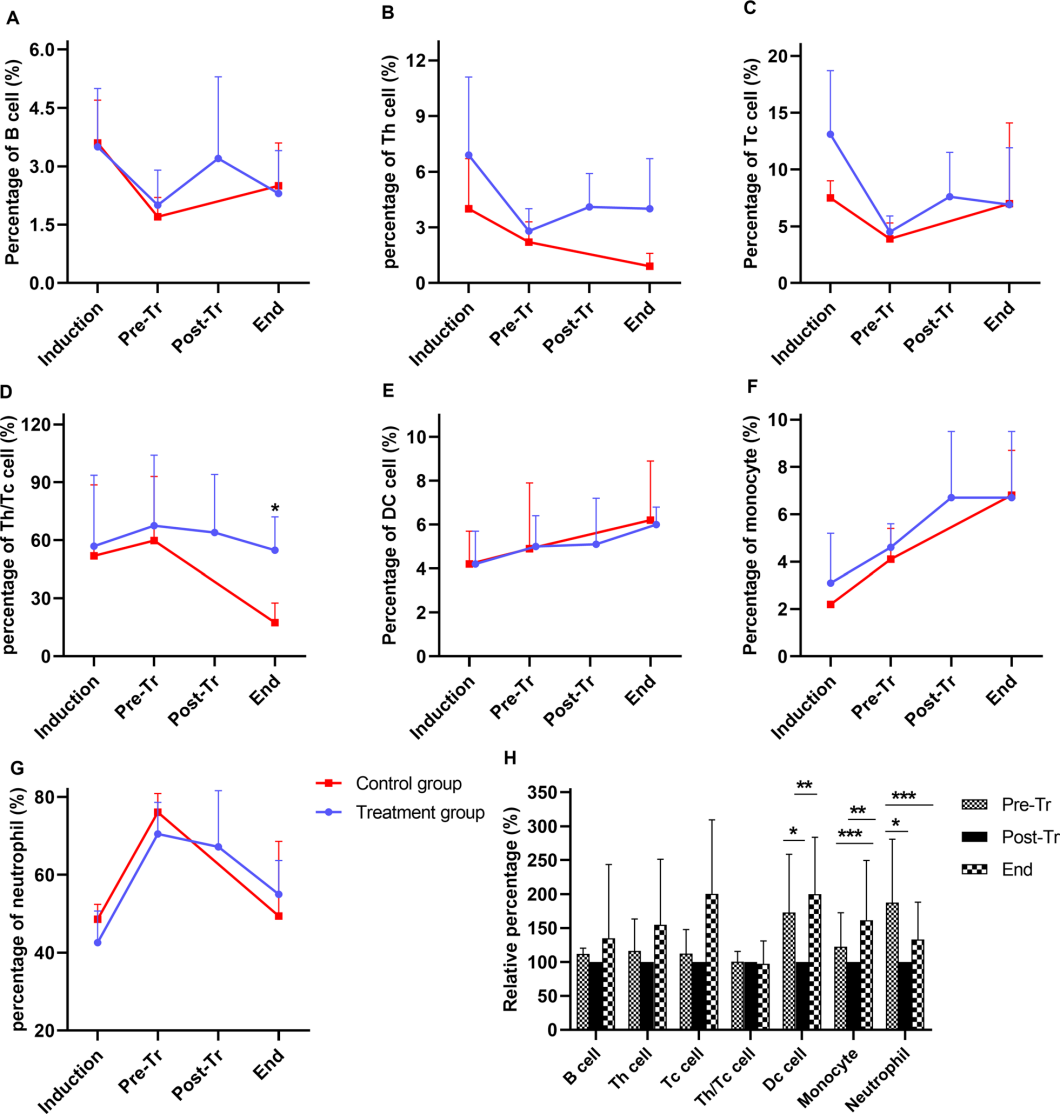
Changes in subgroups of leukocytes. **A** B cells; **B** DCs; **C** Th cells; **D** Tc cells; **E** Th/Tc cells; **F** monocytes; **G** neutrophils; **H** changes before and after treatment. Among Fig. **A**–**G**, (*) stands for statistical significance between the two groups (P < 0.05). In Fig. **H**, (*) stands for statistical significance between Pre-Pr and Post-Dr points (P < 0.05), (**) stands for statistical significance between End and Post-Tr points (P < 0.05), and (***) stands for statistical significance between Pre-Tr and End points (P < 0.05). (Induction, baseline and sepsis induction; Pre-Tr, 12 h after sepsis induction and before treatment; Post-Tr, the end of treatment; End, 24 h after treatment and time before euthanasia)

After the induction of sepsis, with a progressive increase in leukocyte count, the subgroups of leukocytes presented divergent patterns: in the control group, the counts of DCs, monocytes and neutrophils increased, while the counts of Th cells declined; in the treatment group, the change trends were obviously flattened (Fig. 5). The numbers of B cells, Tc cells, DC cells, monocytes and activated neutrophils were lower and that of Th cells was higher in the treatment group than in the control group at 24 h post-treatment, although nonsignificantly. The ratios of Th cells and Tc cells (Th/Tc) were significantly higher in the treatment group than in the control group (P = 0.015) (Fig. 5E).

The treatment had different effects on subgroups of leukocytes: no change was found for B cells, Th cells, or Tc cells, but a significant reduction of DC cells and neutrophils occurred with treatment. After treatment, DC cells and monocytes rebounded after 24 h (P < 0.05) (Fig. 5H).

## Discussion

This preliminary study demonstrated that extracorporeal cellular apheresis therapy yields an immunomodulatory effect accompanied by clinical improvement of systemic hemodynamics and oxygenation status in a peritonitis-induced septic porcine model, which to our knowledge is the first study to use the extracorporeal centrifugation technique in sepsis therapy.

In this study, we established a sepsis model in domestic pigs by intraperitoneal injection of a suspended solution containing previously incubated autologous feces (1.0 g/kg). This model has an identical size, endotoxin sensitivity, tissue antigenicity and immune response as that in human beings and is suitable for investigating the effect of extracorporeal blood therapy in animal models [19]. Unlike other models using a single infusion of specific live bacteria [20, 21] or endotoxin [22] to induce a transient reaction other than a sustained process, we used a peritonitis-induced sepsis model to mimic polymicrobial and progressive characteristics of clinical situations, most importantly, with an appropriate sepsis severity to avoid rapid death within the first 6 to 12 h due to an overly serious condition [23, 24] and to guarantee a sufficient survival time for the completion of the study. As reported in some experiments, an observed time that is too short may not be enough to obtain a full view of sepsis at the end of the investigation [25, 26]. Notably, we administered the intervention 12 h after the induction of sepsis, which more closely conforms to the clinical real world, rather than before or immediately after the induction of sepsis, as reported in some studies [21, 25]. Since time is needed for the treatment to produce the therapeutic effect, we also spared a 24 h time window to observe the effect of the treatment.

Organ dysfunction is the clinical feature of sepsis, involving hypotension, acute respiratory distress syndrome, disseminated intravascular coagulation and so on [27], and the cumulative effect of organ dysfunction is the strongest predictor of mortality [28]. We observed that after 12 h of sepsis induction, animals showed a decrease in PaO2 and PaO2/FiO2 and an increase in lactate levels, and the trend was further obvious if not treated, which indicated lung injury. After 18 h of sepsis induction, blood pressure showed a downward trend, which is an indication of circulatory disorders. At the 12 h time point, there was a significant difference in the pH value between the control and treatment groups; however, in view of clinical practice, the pH difference of 0.1 units had no clinical significance, and the model of the two groups was consistent. Organ dysfunction is a complicated pathophysiologic process and is a result of the joint action of multiple responses to inflammation, one of which is immune dysregulation [29].

Immune dysfunction of both the native and adaptive immune systems is thought to be the essence of the pathophysiology of sepsis [3]. In the traditional view, sepsis is a process that shifts from initial proinflammatory reactions to subsequent anti-inflammatory reactions, manifested as an initial significant increase in peripheral blood leukocytes, especially neutrophils and monocytes, immediately following lymphopenia [30]. However, in this study, we observed a significant increase in neutrophils and monocytes and a decrease in lymphocytes (including B cells, Th cells and Tc cells) in the initial 12 h of sepsis, although without a significant difference. As in the profile of serum cytokines, a simultaneous proinflammatory and anti-inflammatory response was present. This conforms to the characteristics of the immune response in sepsis recently described by Xiao WZ et al. [31], in which they revealed a simultaneous rapid and sustained upregulation of genes involved in the innate immune and pro/anti-inflammatory response, as well as a downregulation of genes involved in adaptive immunity in patients suffering from severe trauma or burn injury.

Leukocytapheresis is a technique using extracorporeal centrifugation to separate blood constituents according to their density, and we used the MNC program in this study to remove blood MNCs from the circulation, with the expectation of attenuating the negative reactions of sepsis. As revealed by the results, various kinds of blood-formed elements were removed by LCAP, including B cells, CD4 + T cells, CD8 + T cells, DC cells, monocytes, platelets, and neutrophils, all of which are involved in sepsis-induced innate and adaptive immune derangements relevant to sepsis recovery and survival [3]. The cell selectivity (different cells were cleared in different amounts and ratios) may have been related to the cell density because LCAP stratified the blood components by centrifugal force according to the specific gravity of different cells. The effects of LCAP on sepsis as revealed by the study should be a wide-spectrum and bidirectional effect on sepsis-activated immune cells, manifested as a flattening of the change trends of various immune cells induced by sepsis, accompanied by improvement of two vital clinical conditions of sepsis, that is, hemodynamics and oxygenation.

An interesting phenomenon shown in our experiment is that a considerable quantity of neutrophils was removed by LCAP via the MCN program because the density difference between granulocytes and mononuclear cells will generally make them appear in different layers under density gradient centrifugation. This may be explained by the fact that abnormally dense neutrophils, also called low-density neutrophils (LDNs), may be present in the blood of patients with infections, cancer, pregnancy and autoimmune diseases [32]. However, thus far, it is difficult to distinguish LDNs from normal-density neutrophils (NDNs) by common markers such as CD11b, CD15, CD66b and CD33, although a difference in the expression level may exist [33]. We used CD11b as a marker [34] to show activated neutrophils accounting for 59.6% of the collection solution by LCAP and a lower level of them in circulation after LCAP, but with a significantly higher level of MPO, an indicator of the antimicrobial activity of neutrophils [35]. This result may imply that elimination of overactivated neutrophils from the circulation does not impair but rather enhances the antimicrobial capacity of the body. Another interesting phenomenon is that all the cells rebounded after 24 h of treatment, although only the rebounding of DC cells and monocytes was statistically significant. The apoptosis of DC cells engages in the immune suppression of sepsis [36]. Multiple reports have shown that prevention of sepsis-induced DC apoptosis or augmentation of DC function enhances sepsis survival [37, 38]. The long-term effect (at least 24 h) of LCAP increases the number of DC cells, and cell function needs to be explored in the future.

Other methods have also been reported for the extracorporeal removal of activated immune cells. One of them is a selective cytopheretic device (SCD) with regional citrate anticoagulation, which was reported to absorb activated leukocytes (mainly neutrophils) and improve the 60-day mortality when the postfilter ionized calcium (iCa) level was  ≤ 0.4 mmol/L [39]. Cellsorba is a device with polyethylenephtarate fibers that unselectively capture monocytes, granulocytes, and lymphocytes and is used for the treatment of inflammatory bowel disease in Japan and Europe, but its effects remain unclear [40]. These are all complicated extracorporeal circulation techniques, requiring a high dose of anticoagulants, central venous catheterization to afford a high blood flow rate, and a specialized team to handle the complex circuits, which obviously hinder their clinical applications regardless of the clinical effects. In contrast, extracorporeal centrifugal cellular apheresis, as a mature technique with low blood flow rates of 40–60 ml/min, a simple filter-free circuit and low clotting risk, may be worth further exploring for its application in the treatment of sepsis.

The obvious limitations of our study should be stated. First, the sepsis model of the study was not very severe, with modest pathological lesions in organs and a survival time beyond 48 h in order to guarantee the accomplishment of the study. Second, no antibiotics, as recommended by consensus [2], were used for the treatment of sepsis; third, only one treatment at a specific time point of sepsis was administered in the study, considering that sepsis is a sustained inflammatory response process, and one shot is obviously not enough. Finally, no hard endpoints were considered in the study, and consequently, further studies are needed to investigate the effect of LCAP on sepsis mortality.

## Conclusions

In conclusion, this preliminary study using LCAP to treat septic pigs revealed an immunomodulatory effect with improvement of vital clinical conditions. However, further studies are still needed to verify its role in improving the mortality of sepsis.

## Data Availability

All data generated or analyzed during this study are included in this published article.

## Abbreviations

LCAP: Leukocytapheresis
MAP: Mean arterial blood pressure
HVHF: High-volume hemofiltration
WBC: White blood cells
ACD-A: Acid citrate dextrose solution A
TBV: Total blood volume
CRP: C-reactive protein
PCT: Procalcitonin
MPO: Myeloperoxidase
LDN: Low-density neutrophils
NDNs: Normal density neutrophils
SCD: Selective cytopheretic device
iCa: Ionized calcium
